# Comparison of overall survival and disease-free survival for breast-conserving surgery and mastectomy in breast cancer patients receiving neoadjuvant therapy: a matched case-control study from two institutions

**DOI:** 10.3389/fonc.2025.1681937

**Published:** 2026-01-05

**Authors:** Xueqian Du, Senyan Zhang, Yilu Li, Yue Li, Yueqing Feng

**Affiliations:** 1 Head, Neck and Breast Ward 2, Department of Surgical Oncology, Xinxiang Central Hospital, Xinxiang, China; 2 Department of Thyroid and Breast Surgery, Peking University First Hospital, Beijing, China

**Keywords:** breast cancer, breast-conserving surgery, mastectomy, neoadjuvant therapy, survival

## Abstract

**Background:**

Neoadjuvant therapy (NAT) has been increasingly promoted for treating early-stage breast cancer (BC), which significantly improves the adoption of breast-conserving surgery (BCS). However, concerns related to the oncological safety of BCS versus mastectomy remain unelucidated. The present study compared survival outcomes between patients treated with BCS and those treated with mastectomy after NAT through stratified analyses.

**Methods:**

The study included female BC patients who underwent radical surgery after NAT at the Peking University First Hospital and Cancer Hospital of Chinese Academy of Medical Sciences from January 2013 to December 2021. Propensity score matching (PSM) was used to minimize the selection bias. Overall survival (OS) and disease-free survival (DFS) were compared between patients receiving BCS and mastectomy.

**Results:**

A total of 994 patients were enrolled, including 285 patients treated with BCS and 709 patients treated with mastectomy. Following PSM, patients were assigned to the BCS (n = 258) and mastectomy (n = 258) groups; these two groups were well balanced regarding clinical and pathological characteristics. The 5-year OS rate (90.5% *vs*. 95.8%, *P* = 0.535) and DFS rate (86.3% *vs*. 86.9%, *P* = 0.648) of the mastectomy group were identical to those of the BCS group in the matched cohort. Stratified analysis revealed that mastectomy was an independent adverse prognostic factor for OS (hazard ratio [HR]: 2.158, 95% CI: 1.254–4.954, *P* = 0.034) and DFS (HR: 2.914, 95% CI: 1.713–5.422, *P* = 0.010) in patients with positive lymph nodes. Additionally, age-based stratification showed that mastectomy was an independent prognostic factor for DFS in BC patients aged > 40 years (HR: 2.471, 95% CI: 1.082–5.643, *P* = 0.022).

**Conclusion:**

BCS does not affect OS and DFS in BC patients treated with NAT. However, it should be noted that BCS provides a substantial survival benefit as compared to mastectomy in patients with clinically positive lymph nodes and those aged > 40 years.

## Introduction

Several randomized controlled trials (RCTs) have shown that the prognosis of breast-conserving surgery (BCS) plus radiotherapy (RT) is similar to that of mastectomy in patients with early-stage breast cancer (BC) (1, 2), and certain patients treated with BCS + RT even showed better survival benefits (3–8). In the past two decades, neoadjuvant therapy (NAT) has been increasingly recommended and rapidly promoted for treating early-stage BC. On the one hand, NAT can decrease the primary tumor volume, improve the pathological complete response (pCR) rate, and increase the possibility of undergoing BCS. On the other hand, compared to the adjuvant setting, the efficacy of the new regimen can be tested and determined more rapidly and accurately in a NAT setting (9). Because BCS after NAT is generally performed in patients with large tumors who are deemed unsuitable to undergo BCS at the time of diagnosis, it is essential to investigate the oncological safety of BCS after NAT in these patients. Regarding the NAT setting, a meta-analysis by the Early Breast Cancer Trialists’ Collaborative Group on the prognosis of NAT showed higher local recurrence risk for BCS (10). However, some studies have reported that the survival outcomes of patients undergoing BCS + RT treatment is equal to or better than that of patients undergoing mastectomy after NAT (11–13). The inconsistency in the results may be related to differences in study duration, postoperative RT delivery, and treatment decisions, different margin control in BCS, and this contradiction should be balanced and resolved. Moreover, it is not feasible to make a general comparison between these two surgical procedures, and a detailed analysis should be conducted based on various preoperative characteristics such as age, tumor location, T stage, node status, presence of ductal carcinoma *in situ* (DCIS), and degree of tumor regression. Therefore, the present study aimed to compare the prognostic differences of BC patients treated with NAT who underwent BCS with those of patients who underwent mastectomy and to conduct stratified analysis of patients with different clinical characteristics to refine the indications of BCS after NAT.

## Patients and methods

### Patients

This was a two-center, retrospective study based on the data collected from a prospective institutional database. Female BC patients who underwent radical surgery after NAT at the Peking University First Hospital and Xinxiang Central hospital from January 2013 to December 2021 were identified. The inclusion criteria were as follows: (1) age: 18–75 years; (2) clinical stage: T1–3 N0–2 M0; and (3) RT was performed after BCS. The exclusion criteria were as follows: (1) distant metastases; (2) less than 4 cycles of NAT; (3) incomplete data; (4) diagnosis of occult BC, bilateral BC, or inflammatory BC; and (5) previous history of other malignancies. This study received ethical approval from the ethics committee of the Peking University First Hospital and Xinxiang Central hospital (approval number: 2023-479-002), and all enrolled patients provided written informed consent.

### Diagnosis and treatment

Data on patient demographic characteristics, tumor features, and treatment details were collected prospectively from the institutional database. The estrogen receptor (ER) and progesterone receptor (PR) expression levels were evaluated with the Allred score (14). The ER/PR status was considered positive for tumor nuclear staining of ≥1%. Both ER and PR are hormonal receptors (HRs). HR-positive represents BC positive for ER and/or PR, while HR-negative implies BC negative for ER and PR. HER2(2+) and fluorescence *in situ* hybridization (FISH)-positive or HER2(3+) was defined as HER2-positive BC, and HER2(2+) and FISH-negative or HER2 (1+ or 0) was confirmed as HER2-negative BC (15). TNM staging was based on the eighth edition of the Cancer Staging Manual of the American Joint Committee on Cancer (16).

All patients need to undergo breast MR before NAT to assess the extent of the tumor lesion and determine whether there are contraindications for BCS. NAT regimens were determined according to the guidelines of Chinese Society of Clinical Oncology (17) and recommendations from the multidisciplinary tumor board. Patients with triple-negative breast cancer and HR-positive/HER2-negative breast cancer received anthracycline plus paclitaxel for a total of 6 cycles. HER2-positive BC patients received anti-HER2 targeted therapy (Trastuzumab or trastuzumab combined with pertuzumab) in addition to chemotherapy (paclitaxel in combination with carboplatin). After completion of NAT, radical surgery involving either BCS or mastectomy was performed. For the BCS procedure, surgeons aimed to remove 0.5–1.0 cm of the normal tissue along with the primary tumor tissue; once the surgical margin was positive (DCIS was less than 2 mm from the margin or invasive cancer was adjacent to the margin) after BCS, re-excision (repeat BCS or mastectomy) was indicated. Regarding axillary management, sentinel lymph node biopsy (SLNB) was performed for clinically node-negative patients prior to NAT, and axillary lymph node dissection was performed for clinically node-positive patients or patients with a positive SLNB result. pCR was defined as the absence of invasive carcinoma components in breast lesions and the presence of residual ductal carcinoma *in situ* components (ypT0/is) (18).

### Follow-up

All patients were recommended to undergo outpatient follow-up every 3 months in the first 2 years after surgery and then every 6 months until 5 years from surgery. The follow-up deadline was December 31, 2023. Initial and follow-up examination items included mammography, breast ultrasound imaging, magnetic resonance imaging (MRI), chest radiography, laboratory tests, and clinical examination. All patients received adjuvant RT after BCS. For patients undergoing NAT, the need for adjuvant RT was decided based on the initial TNM stage during disease course. Women who had positive lymph nodes or T3 tumor and received mastectomy were treated with RT recommended to the chest wall and regional lymph node. For patients undergoing BCS and those undergoing total mastectomy, the radiotherapy irradiation sites are the whole breast and the chest wall, respectively. For patients with positive lymph nodes, regional lymph nodes are irradiated (lymph nodes in the armpit, internal mammary, and supraclavicular and subclavicular regions). For patients undergoing BCS, the total radiotherapy dose is 50Gy/25 times. For the area where the tumor is located, the irradiation dose is increased by an additional 10-16Gy, for a total of 5–8 times. The total radiotherapy dose for patients undergoing mastectomy was 42.5Gy/16 times. Patients with HR-positive BC and HER2-positve BC received endocrine therapy and anti-HER2 therapy after surgery, respectively. The endpoint of this study was 5-year overall survival (OS) rate and disease-free survival (DFS) rate.

### Statistical analysis

To minimize the disproportionate impact of variables other than the surgical method on the study results, a propensity score matching (PSM) (nearest neighbor ratio of 1:1 without replacement and a caliper with of 0.02) was performed. The matched variables were age, tumor location, clinical T stage, clinical node status, tumor subtype, tumor grade, presence of DCIS in pre-NAT biopsy, Ki-67 index, and pCR. Adjuvant RT was not used as a variable for matching.

We conducted t-tests and chi‐square tests to compare continuous variables and categorical variables between the groups, respectively. Survival rates (OS and DFS) were analyzed with Kaplan-Meier survival curves and compared between the BCS and mastectomy groups with the log-rank test. Statistically significant variables in the univariate analysis and the chosen surgical procedure (BCS or mastectomy) were subsequently tested by multivariate analysis through a Cox regression model, and the effect of each variable was assessed based on hazard ratio (HR) and 95% confidence interval (95% CI). Because age, clinical T stage, clinical node status, tumor subtype, tumor grade, presence of DCIS in pre-NAT biopsy, Ki-67 index, and pCR influenced the choice of treatment modality and survival prognosis of patients, we performed stratified analyses based on these abovementioned variables. A *P*-value of <0.05 was considered statistically significant.

## Results

### Baseline characteristics

**Figure 1:**
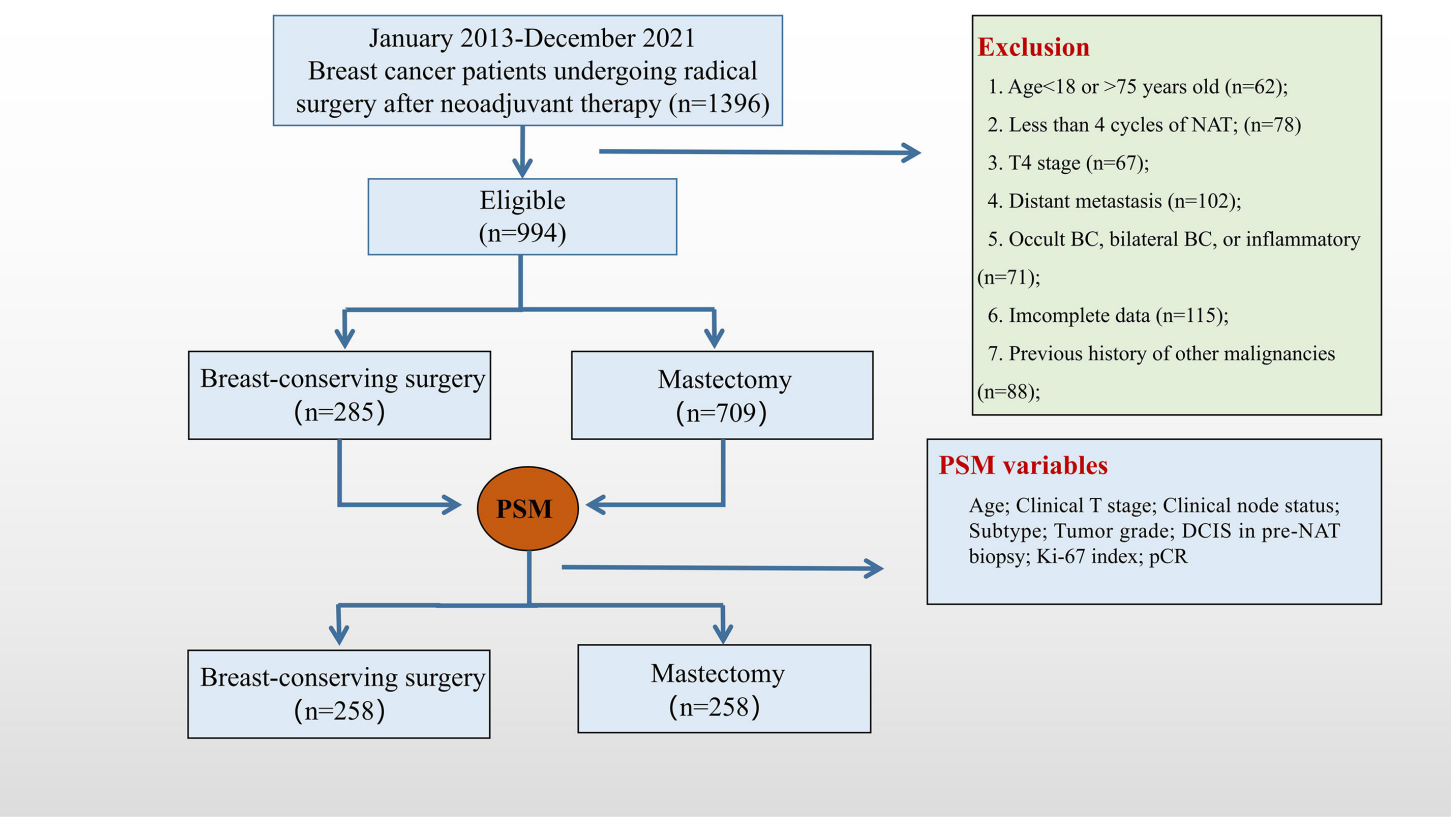
Study flowchart.

From January 2013 to December 2021, a total of 1396 female BC patients received radical surgery after NAT at the Peking University First Hospital and Cancer Hospital of Chinese Academy of Medical Sciences. Of these 1396 patients, 994 patients were eligible for the analysis and were included in this study. Among the 994 patients, 285 and 709 patients were treated with BCS and mastectomy, respectively. By using the PSM method, 258 matched pairs were selected and stratified into the BCS group (n = 258) and the mastectomy group (n = 258) (**Figure 1**).

The clinical and pathological characteristics of the patients before and after PSM between the groups are shown in **Table 1**. Before PSM, compared to the mastectomy group, patients in the BCS group were significantly younger (46.0 ± 10.7 *vs*. 51.2 ± 9.8 years, *P* < 0.001), and the proportion of patients aged ≤ 40 years was significantly higher in the BCS group than in the mastectomy group (33.0% *vs*. 15.7%, *P* < 0.001). Furthermore, compared to the BCS group, the mastectomy group showed more centrally located tumors (8.6% *vs*. 3.2%, *P* = 0.002). Additionally, the proportion of patients with clinical T3 stage (15.2% *vs*. 6.3%, *P* < 0.001) and positive lymph nodes (68.5% *vs*. 49.5%, *P* < 0.001) was significantly higher in the mastectomy group than in the BCS group. All patients in the BCS group received adjuvant RT, while 64.6% of the patients in the mastectomy group received adjuvant RT; this difference was statistically significant (*P* < 0.001). After PSM (except for adjuvant RT), the BCS and mastectomy groups were well balanced in all the abovementioned variables (*P* > 0.05).

**Table 1:** The clinical and pathological characteristics before and post matching.

Variables	Total cohort			Matched cohort		
	BCS (n=285)	Mastectomy (n=709)	*P*	BCS (n=258)	Mastectomy (n=258)	*P*
Age (mean±SD, years)	46.0±10.7	51.2±9.8	<0.001	48.1±9.9	49.0±10.9	0.403
Age (years)			<0.001			0.921
≤40	94 (33.0)	111 (15.7)		69 (26.7)	70 (27.1)	
>40	191 (67.0)	598 (84.3)		189 (73.3)	188 (72.9)	
Tumor location			0.002			0.648
Central type	9 (3.2)	61 (8.6)		9 (3.5)	11 (4.3)	
Non-central type	276 (96.8)	648 (91.4)		249 (96.5)	247 (95.7)	
Clinical T stage (pre-NAT)			<0.001			0.589
T1-T2	267 (93.7)	601 (84.8)		243 (94.2)	240 (93.0)	
T3	18 (6.3)	108 (15.2)		15 (5.8)	18 (7.0)	
Clinical node status (pre-NAT)			<0.001			0.724
Positive	141 (49.5)	486 (68.5)		136 (52.7)	140 (54.3)	
Negative	144 (50.5)	223 (31.5)		122 (47.3)	118 (45.7)	
Subtype			0.253			0.436
HR+/HER2-	81 (28.5)	205 (28.9)		75 (29.1)	63 (24.4)	
HR+/HER2+	69 (24.2)	173 (24.4)		63 (24.4)	63 (24.4)	
HR-/HER2+	44 (15.4)	141 (19.9)		44 (17.1)	57 (22.1)	
HR-/HER2-	91 (31.9)	190 (26.8)		76 (29.4)	75 (29.1)	
Tumor grade (pre-NAT)			0.406			0.591
G1-G2	114 (40.0)	304 (42.9)		108 (41.9)	102 (39.5)	
G3	171 (60.0)	405 (57.1)		150 (58.1)	156 (60.5)	
DCIS in pre-NAT biopsy			0.824			0.768
Presence	78 (27.4)	199 (28.1)		73 (28.3)	70 (27.1)	
Absence	207 (72.6)	510 (71.9)		185 (71.7)	188 (72.9)	
Ki67 index (pre-NAT)			0.359			0.824
<20%	12 (4.2)	40 (5.6)		10 (3.9)	11 (4.3)	
≥20%	273 (95.8)	669 (94.4)		248 (96.1)	247 (95.7)	
pCR			0.647			0.701
Yes	78 (27.4)	184 (26.0)		76 (29.5)	80 (31.0)	
No	207 (72.6)	525 (74.0)		182 (70.5)	178 (69.0)	
Adjuvant radiotherapy			<0.001			<0.001
Yes	285 (100.0)	458 (64.6)		258 (100.0)	125 (48.4)	
No	0 (0)	251 (35.4)		0 (0)	133 (51.6)	

BCS, breast-conserving surgery; NAT, neoadjuvant therapy; DCIS, ductal carcinoma in-situ; pCR, pathological complete response.

### Survival analysis

**Figure 2:**
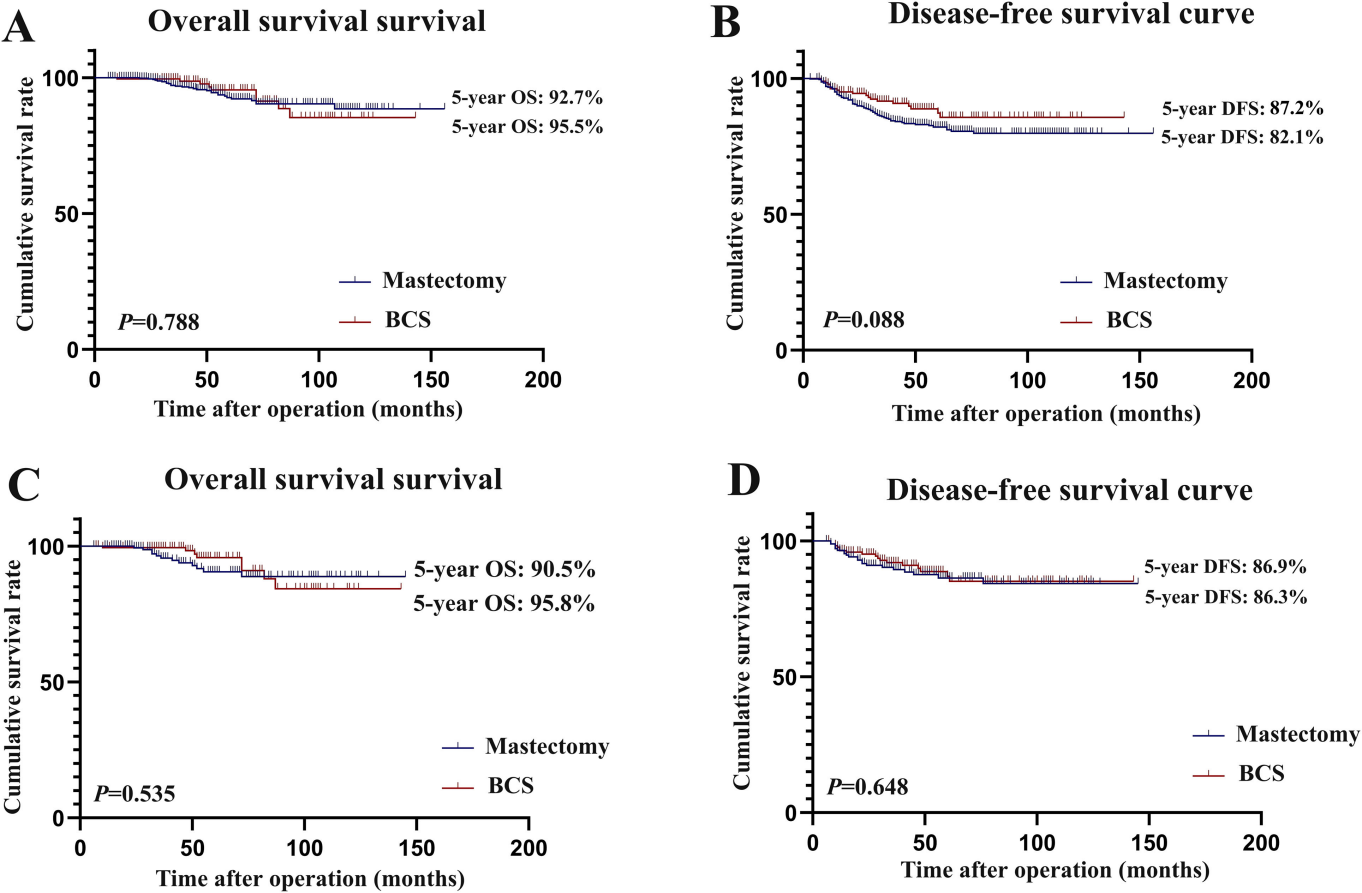
Overall survival and disease-free survival curve of patients in the breast-conserving surgery group and the mastectomy group before and after matching. **(A)** overall survival before matching; **(B)** disease-free survival before matching; **(C)** overall survival after matching; **(D)** disease-free survival after matching.

The median follow-up period for all the enrolled patients was 61.0 and 63.0 months before and after PSM, respectively. Before PSM, both BCS and mastectomy groups had similar 5-year OS rates (95.5% *vs*. 92.7%, *P* = 0.788) (**Figure 2A**), and the 5-year DFS rate was higher in the BCS group than in the mastectomy group (87.2% *vs*. 82.1%, *P* = 0.088) (**Figure 2B**); however, the difference was not statistically significant. After PSM, the estimated 5-year OS rates were 90.5% and 95.8% in the mastectomy and BCS groups, respectively (*P* = 0.535) (**Figure 2C**), and the 5-year DFS rates were 86.3% and 86.9% in the mastectomy and BCS groups, respectively (*P* = 0.648) (**Figure 2D**).

The results of univariate and multivariate analyses of prognostic factors influencing the OS and DFS of the overall cohort are presented in **Table 2**. In the univariate analysis, clinical T stage, clinical node status, tumor subtype, and pCR significantly affected both OS and DFS (*P* < 0.05). In the multivariate analysis, clinical T3 stage (HR: 4.876, 95% CI: 2.463–9.652, *P* < 0.001), positive lymph node (HR: 2.545, 95% CI: 1.039–6.234, *P* = 0.041), and HR-/HER2- status (HR: 3.701, 95% CI: 1.521–7.172, *P* = 0.005) were the independent prognostic factors for OS. Additionally, in the multivariate analysis, clinical T3 stage (HR: 2.666, 95% CI: 1.670–4.255, *P* < 0.001), positive lymph node (HR: 2.596, 95% CI: 1.502–4.486, *P* < 0.001), HR-/HER2- status (HR: 3.005, 95% CI: 1.520–6.912, *P* < 0.001), and pCR (HR: 1.939, 95% CI: 1.114–3.372, *P* = 0.019) were the independent prognostic factors for DFS.

**Table 2:** Univariate and multivariate analysis for overall survival and disease-free survival in overall cohorts.

Variables	Overall survival				Disease-free survival			
	Univariate analysis		Multivariate analysis		Univariate analysis		Multivariate analysis	
	HR(95%CI)	*P*	HR(95%CI)	*P*	HR(95%CI)	*P*	HR(95%CI)	*P*
Age at operation (>40 years vs ≤40 years)	0.853 (0.390-1.868)	0.691			0.745 (0.469-1.186)	0.215		
Tumor location (central type vs non-central type )	1.533 (0.690-6.328)	0.572			1.892 (0.580-8.901)	0.470		
Clinical T stage (T3/T1-T2)	5.389 (2.793-10.398)	<0.001	4.876 (2.463-9.652)	<0.001	3.130 (1.979-4.951)	<0.001	2.666 (1.670-4.255)	<0.001
Clinical node status (positive/negative)	3.045 (1.270-7.300)	0.013	2.545 (1.039-6.234)	0.041	3.027 (1.768-5.184)	<0.001	2.596 (1.502-4.486)	<0.001
Subtype								
HR+/HER2-								
HR+/HER2+	2.013 (0.520-6.301)	0.572	2.590 (0.599-8.891)	0.590	2.181 (0.769-7.911)	0.275	1.281 (0.721-6.852)	0.389
HR-/HER2+	2.402 (0.862-5.212)	0.182	3.011 (0.755-7.210)	0.251	2.581 (0.914-6.019)	0.103	2.021 (0.842-5.301)	0.192
HR-/HER2-	2.589 (1.290-5.320)	<0.001	3.701 (1.521-7.172)	0.005	3.501 (2.020-8.592)	<0.001	3.005 (1.520-6.912)	<0.001
Tumor grade (G3/G1-G2)	1.319 (0.672-2.591)	0.421			1.031 (0.684-1.554)	0.884		
DCIS in pre-NAT biopsy (presence/absence)	0.922 (0.446-1.905)	0.827			0.728 (0.448-1.183)	0.200		
Ki67 index (≥20%/<20%)	2.093 (0.287-15.271)	0.466			1.362 (0.596-3.114)	0.464		
pCR (no/yes)	2.629 (1.024-6.750)	0.045	2.252 (0.874-5.807)	0.093	2.135 (1.229-3.708)	0.007	1.939 (1.114-3.372)	0.019
Types of surgery (mastectomy vs BCS)	1.408 (0.523-2.349)	0.788	1.698 (0.718-2.534)	0.371	1.743 (0.932-2.552)	0.088	1.451 (0.687-1.929)	0.594

DCIS, ductal carcinoma in-situ; pCR, pathological complete response.

### Subgroup analysis for prognosis

To investigate the prognostic differences between patients with various clinical and pathological characteristics who received BCS and mastectomy, we subdivided them according to different variables (age, tumor location, clinical T stage, clinical node status, tumor subtype, tumor grade, presence of DCIS in pre-NAT biopsy, Ki-67 index, and pCR) and conducted stratified multivariate Cox proportional risk regression analysis on the effects of the surgical procedure (BCS and mastectomy) on OS (**Table 3**) and DFS (**Table 4**).

**Table 3:** Cox proportional risk regression of overall survival for patients in different subgroup who underwent BCS or mastectomy.

Variables	Types of surgery	Univariate analysis		Multivariate analysis	
		HR (95%CI)	*P*	HR (95%CI)	*P*
Age (years)^a^					
≤40	Mastectomy vs BCS	1.136 (0.270-4.785)	0.862	0.383 (0.116-3.046)	0.368
>40	Mastectomy vs BCS	1.743 (0.837-6.988)	0.115	2.381 (0.771-9.542)	0.103
Tumor location^b^					
Central type	Mastectomy vs BCS	0.843 (0.489-1.992)	0.190	0.759 (0.479-2.301)	0.285
Non-central type	Mastectomy vs BCS	1.729 (0.420-2.841)	0.585	1.901 (0.459-2.640)	0.391
Clinical T stage^c^					
T1-T2	Mastectomy vs BCS	1.089 (0.689-2.643)	0.400	1.340 (0.662-2.567)	0.329
T3	Mastectomy vs BCS	1.845 (0.202-11.841)	0.675	1.976 (0.177-14.047)	0.684
Clinical node status^d^					
Positive	Mastectomy vs BCS	2.312 (1.415-6.471)	0.019	2.158 (1.254-4.954)	0.034
Negative	Mastectomy vs BCS	1.039 (0.463-2.336)	0.925	0.714 (0.303-1.682)	0.441
Subtype^e^					
HR+/HER2-	Mastectomy vs BCS	1.875 (0.458-9.393)	0.343	2.310 (0.359-14.877)	0.378
HR+/HER2+	Mastectomy vs BCS	1.142 (0.465-2.804)	0.773	1.132 (0.447-2.869)	0.793
HR-/HER2+	Mastectomy vs BCS	1.223 (0.146-10.217)	0.852	0.591 (0.052-6.771)	0.672
HR-/HER2-	Mastectomy vs BCS	1.838 (0.617-5.477)	0.275	0.316 (0.086-1.165)	0.084
Tumor grade^f^					
G1-G2	Mastectomy vs BCS	1.085 (0.298-3.946)	0.901	1.645 (0.644-3.882)	0.566
G3	Mastectomy vs BCS	1.776 (0.426-2.719)	0.877	2.024 (0.605-3.384)	0.682
DCIS in pre-NAT biopsy^g^					
Presence	Mastectomy vs BCS	0.507 (0.143-1.801)	0.294	0.783 (0.293-3.650)	0.564
Absence	Mastectomy vs BCS	1.785 (0.600-4.191)	0.353	1.954 (0.416-3.199)	0.783
Ki67 index^h^					
<20%	Mastectomy vs BCS	2.412 (0.421-18.224)	0.856	1.282 (0.234-12.55)	0.939
≥20%	Mastectomy vs BCS	1.122 (0.527-2.386)	0.765	1.927 (0.728-3.610)	0.432
pCR^i^					
Yes	Mastectomy vs BCS	1.892 (0.420-4.921)	0.419	2.941 (0.401-5.024)	0.906
No	Mastectomy vs BCS	0.940 (0.435-2.032)	0.875	0.853 (0.239-1.276)	0.165

BCS, breast-conserving surgery; DCIS, ductal carcinoma in-situ; pCR, pathological complete response.

a Multivariable analysis adjusted for tumor location, clinical T stage, clinical node status, subtype, tumor grade, DCIS in pre-NAT biopsy, Ki-67 index, and pCR.

b Multivariable analysis adjusted for age, clinical T stage, clinical node status, subtype, tumor grade, DCIS in pre-NAT biopsy, Ki-67 index, and pCR.

c Multivariable analysis adjusted for age, tumor location, clinical node status, subtype, tumor grade, DCIS in pre-NAT biopsy, Ki-67 index, and pCR.

d Multivariable analysis adjusted for age, tumor location, clinical T stage, subtype, tumor grade, DCIS in pre-NAT biopsy, Ki-67 index, and pCR.

e Multivariable analysis adjusted for age, tumor location, clinical T stage, clinical node status, subtype, DCIS in pre-NAT biopsy, Ki-67 index, and pCR.

f Multivariable analysis adjusted for age, tumor location, clinical T stage, clinical node status, subtype, DCIS in pre-NAT biopsy, Ki-67 index, and pCR.

g Multivariable analysis adjusted for age, tumor location, clinical T stage, clinical node status, subtype, tumor grade, Ki-67 index, and pCR.

h Multivariable analysis adjusted for age, tumor location, clinical T stage, clinical node status, subtype, tumor grade, DCIS in pre-NAT biopsy, and pCR.

i Multivariable analysis adjusted for age, tumor location, clinical T stage, clinical node status, subtype, tumor grade, DCIS in pre-NAT biopsy, and Ki-67 index.

**Table 4:** Cox proportional risk regression of disease-free survival for patients in different subgroup who underwent BCS or mastectomy.

Variables	Types of surgery	Univariate analysis		Multivariate analysis	
		HR (95%CI)	*P*	HR (95%CI)	*P*
Age (years)^a^					
≤40	Mastectomy vs BCS	1.130 (0.582-3.042)	0.499	0.753 (0.295-1.924)	0.554
>40	Mastectomy vs BCS	2.891 (1.311-6.377)	0.009	2.471 (1.082-5.643)	0.022
Tumor location^b^					
Central type	Mastectomy vs BCS	0.740 (0.301-2.522)	0.105	0.682 (0.292-3.529)	0.298
Non-central type	Mastectomy vs BCS	2.158 (0.459-5.992)	0.588	1.801 (0.592-5.891)	0.691
Clinical T stage^c^					
T1-T2	Mastectomy vs BCS	1.205 (0.704-2.063)	0.497	1.105 (0.636-1.920)	0.724
T3	Mastectomy vs BCS	3.298 (0.446-24.408)	0.243	2.809 (0.364-21.701)	0.322
Clinical node status^d^					
Positive	Mastectomy vs BCS	3.403 (1.884-5.545)	0.003	2.914 (1.713-5.422)	0.010
Negative	Mastectomy vs BCS	1.491 (0.884-2.512)	0.134	1.064 (0.789-2.356)	0.466
Subtype^e^					
HR+/HER2-	Mastectomy vs BCS	2.706 (0.949-7.717)	0.063	2.196 (0.723-6.666)	0.165
HR+/HER2+	Mastectomy vs BCS	1.894 (0.970-3.699)	0.062	1.471 (0.744-2.910)	0.268
HR-/HER2+	Mastectomy vs BCS	0.947 (0.264-3.401)	0.933	0.675 (0.177-2.573)	0.565
HR-/HER2-	Mastectomy vs BCS	0.738 (0.319-1.706)	0.477	0.552 (0.234-1.922)	0.434
Tumor grade^f^					
G1-G2	Mastectomy vs BCS	1.398 (0.643-3.042)	0.398	1.064 (0.459-2.464)	0.885
G3	Mastectomy vs BCS	2.259 (0.857-4.212)	0.133	2.040 (0.546-5.422)	0.728
DCIS in pre-NAT biopsy^g^					
Presence	Mastectomy vs BCS	0.729 (0.294-1.806)	0.284	0.534 (0.206-1.383)	0.196
Absence	Mastectomy vs BCS	2.033 (1.095-3.776)	0.025	1.635 (0.856-3.124)	0.136
Ki67 index^h^					
<20%	Mastectomy vs BCS	2.654 (0.591-17.222)	0.692	1.382 (0.321-11.19)	0.810
≥20%	Mastectomy vs BCS	1.462 (0.880-2.429)	0.143	1.968 (0.688-1.982)	0.566
pCR^i^					
Yes	Mastectomy vs BCS	1.558 (0.439-5.527)	0.492	0.913 (0.232-3.593)	0.896
No	Mastectomy vs BCS	2.550 (0.895-4.683)	0.118	1.933 (0.694-2.190)	0.475

BCS, breast-conserving surgery; DCIS, ductal carcinoma in-situ; pCR, pathological complete response.

a Multivariable analysis adjusted for tumor location, clinical T stage, clinical node status, subtype, tumor grade, DCIS in pre-NAT biopsy, Ki-67 index, and pCR.

b Multivariable analysis adjusted for age, clinical T stage, clinical node status, subtype, tumor grade, DCIS in pre-NAT biopsy, Ki-67 index, and pCR.

c Multivariable analysis adjusted for age, tumor location, clinical node status, subtype, tumor grade, DCIS in pre-NAT biopsy, Ki-67 index, and pCR.

d Multivariable analysis adjusted for age, tumor location, clinical T stage, subtype, tumor grade, DCIS in pre-NAT biopsy, Ki-67 index, and pCR.

e Multivariable analysis adjusted for age, tumor location, clinical T stage, clinical node status, subtype, DCIS in pre-NAT biopsy, Ki-67 index, and pCR.

f Multivariable analysis adjusted for age, tumor location, clinical T stage, clinical node status, subtype, DCIS in pre-NAT biopsy, Ki-67 index, and pCR.

g Multivariable analysis adjusted for age, tumor location, clinical T stage, clinical node status, subtype, tumor grade, Ki-67 index, and pCR.

h Multivariable analysis adjusted for age, tumor location, clinical T stage, clinical node status, subtype, tumor grade, DCIS in pre-NAT biopsy, and pCR.

i Multivariable analysis adjusted for age, tumor location, clinical T stage, clinical node status, subtype, tumor grade, DCIS in pre-NAT biopsy, and Ki-67 index.

In stratification based on the clinical node status, surgical procedure was an independent prognostic factor for OS (HR: 2.158, 95% CI: 1.254–4.954, *P* = 0.034) and DFS (HR: 2.914, 95% CI, 1.713–5.422, *P* = 0.010) in patients with a positive lymph node. Additionally, in age-based stratification, surgical procedure was an independent prognostic factor for DFS in BC patients aged > 40 years (HR: 2.471, 95% CI: 1.082–5.643, *P* = 0.022).

## Discussion

NAT is currently widely used for treating early BC, and the accurate localization and subsequent radical resection of the primary tumor after NAT remain a challenge for surgeons. The increased use of MRI, lesion localization markers (such as iodine seeds), multipoint biopsy pathological evaluation, and radiotherapy has greatly reduced the risk of residual tumor and tumor recurrence after NAT followed by BCS, which provides a strong assurance regarding the safety of performing BCS after NAT (12, 19). In the present study, we compared the survival outcomes of BCS versus mastectomy after NAT and found that the 5-year OS rate (90.5% *vs*. 95.8%, *P* = 0.535) and DFS rate (86.3% *vs*. 86.9%, *P* = 0.648) were similar between the mastectomy and BCS groups; however, stratified analyses revealed that patients aged > 40 years or with positive lymph nodes who underwent BCS showed a better prognosis.

Previous studies have shown that the prognosis of BCS is similar to or even better than that of mastectomy in patients receiving NAT (11–13). A Korean Breast Cancer Society study conducted by Gwark et al. demonstrated that patients with operable BC who received NAT and underwent BCS exhibited better DFS rate (87.0% *vs*. 73.1%, *P* < 0.005), distant metastasis-free survival rate (89.5% *vs*. 73.1%, *P* < 0.005), and OS rate (91.8% *vs*. 81.0%, *P* < 0.005) than those who underwent mastectomy after NAT, and these findings remained significant after PSM (11). A study of 612 nonmetastatic BC patients from Netherlands Cancer Registry showed that BCS did not impair OS and DFS (HR: 1.31, 95% CI: 0.81–2.13) for recurrent disease in the mastectomy versus BCS comparison (12). However, an important reason for the difference in prognosis between BCS and mastectomy reported in the literature is inconsistency in the various clinical characteristics (such as TNM stage, tumor subtype, and presence of DCIS) of the patients included in each study (13, 20–24); hence, it is essential to conduct stratified analysis to explore the benefits of both surgical procedures.

The oncological safety of BCS after NAT in patients with large primary tumors is a cause of concern, particularly in patients with cT3 tumor. Consequently, these patients are frequently excluded from RCTs (11, 25, 26). In the present study, stratified analysis was performed for clinical T stages, and the results showed that the OS (*P* = 0.684) and DFS (*P* = 0.322) of BCS in patients with cT3 tumor after NAT were similar to those of mastectomy. A study conducted by Gwark et al. indicated that BCS + RT after NAT can be a safe and superior option as compared to mastectomy, and the DFS and OS rates were not significantly different among patients with cT1, cT2, and cT3 tumors in the BCS + RT group (11). Shin et al. also reported that BCS after in patients with clinical stage III disease is feasible if the tumor size is smaller than 4 cm after NAT (26). Therefore, cT3 stage before NAT is not a contraindication to BCS. However, it should be noted that, in the present study, patients with a positive lymph node before NAT who underwent BCS had significantly better OS rate (*P* = 0.034) and DFS rate (*P* = 0.010) than those who underwent mastectomy. A study using prospective, provincial database that enrolled 13,914 BC patients revealed that BCS was associated with an improved OS (HR: 1.37, *P* < 0.001) and breast cancer-specific survival (HR: 1.32, *P* < 0.001) (23); this finding was consistent with our results. We suggested that some patients with clinical positive lymph nodes could achieve axillary pCR after NAT, and these patients (particularly those with cT1–T2,N1 staging) may show a neglecting attitude and poor compliance after mastectomy; consequently, they may refuse to undergo adjuvant RT, and many of them eventually show a high risk of recurrence (27). Therefore, for patients with clinical positive lymph nodes, BCS + RT is a better treatment option and is more conducive to the management and implementation of adjuvant RT.

Young BC patients show more aggressive tumor biology and poor differentiation as compared to old BC patients. In previous relevant studies, the proportion of young BC patients was relatively low and often diluted in large cohorts; this made it difficult to accurately explore the oncological safety of the application of surgical approach in patients with different age groups. Orozco et al. studied 15,611 women aged <40 years with primary invasive T1–T2, N0–N1 BC and found that young BC patients showed equivalent OS regardless of the surgical approach (BCS and mastectomy) (24). Wang et al. also reported no significant difference in local recurrence, distant metastasis-free survival, and breast cancer-specific survival between BCS and mastectomy in the young age group (≤40 years). However, in the old age group, BCS was associated with a better distant metastasis-free survival rate as compared to mastectomy (HR: 0.58, 95% CI: 0.38–0.87, *P* = 0.008) (13). Similar to previous studies, the present study found that patients in the old age group (age > 40 years) who received BCS had a significantly better OS rate (HR: 2.158, 95% CI: 1.254–4.954, *P* = 0.034) and DFS rate (HR: 2.914, 95% CI: 1.713–5.422, *P* = 0.010) than those receiving mastectomy; however, a similar difference was not observed in the young age group patients (age ≤ 40 years). This might be because mastectomy involves a more extensive surgical procedure, which suppresses immune response and slows down the postoperative recovery of elderly patients, particularly after NAT, thereby affecting the implementation of the subsequent adjuvant RT. It should be noted that it is not the surgical method that affects the prognosis of specific patients, but rather the different surgical methods that influence the implementation of subsequent treatments. With the improvement of the efficacy of NAT and the increasing number of methods available to identify pCR, some patients can even avoid surgery after NAT. Consequently, the surgical scope and trauma of breast cancer surgery are reducing. Therefore, BCS is a preferable option for elderly patients with BC, if both BCS and mastectomy are feasible.

The most important limitation of the present study is its retrospective nature. Although a prospective database was used, many clinical- and treatment-related details, such as MRI evaluation after NAT, blood vessel invasion, and areas and doses of adjuvant RT, were not accurately available. Additionally, heterogeneity existed in patient populations and treatment strategies, which may have impacted the results. Patients who underwent mastectomy tended to be older and have a larger tumor size than those who underwent BCS; however, this study used PSM to balance the relevant prognostic risk factors between the groups. In addition, the median follow-up was only calculated using a simple median, without the reverse Kaplan-Meier calculation. Moreover, in the subgroup analysis, the CIs for HR are very wide and might be limited by the sample size. Finally, some patients refused to undergo treatment according to the current standards at their own request (such as patients with indications for RT but did not receive RT after mastectomy or patients lost to regular follow-up). However, the number of such patients was very small, which did not significantly affect our results; moreover, the study cohort represented a real-world scenario.

In conclusion, our study demonstrated that both BCS and mastectomy show a similar long-term therapeutic effect after balancing the confounding variables for BC patients receiving NAT. BCS provides better OS and DFS in patients aged >40 years and those with clinical positive lymph nodes; therefore, it seems advisable to encourage all suitable patients with the abovementioned characteristics to undergo BCS rather than mastectomy.

## Data Availability

The raw data supporting the conclusions of this article will be made available by the authors, without undue reservation.
